# Segmented therapies in orthodontics

**DOI:** 10.1186/1746-160X-10-S1-O5

**Published:** 2014-12-12

**Authors:** Timo Peltomäki

**Affiliations:** 1 Oral and Maxillofacial Unit, Tampere University Hospital, Tampere, Finland

## 

Segmental osteotomies offer a versatile option for conventional orthodontic surgery. Basically any single tooth or groups of teeth can be repositioned surgically. Segmental osteotomies have been used to correct bimaxillary protrusion, anterior open bite, transverse maxillary deficiency, distal position of mandibular dentoalveolar process, mandibular anterior crowding and in cleft care for various purposes.

Advantages of using segmental osteotomies include shorter treatment time then moving the teeth orthodontically. The technique makes treatment possible also in cases with reduced anchorage, since surgical repositioning of segments does not place demand in anchorage.

There is generally accepted rule concerning segmental osteotomies: The distance a dentoalveolar segment can be moved surgically is not more than can be done orthodontically. That means that the tooth movement limits presented in the envelope of discrepancy (Proffit 1997) apply equally to dentoalveolar segmental osteotomies and orthodontics. The main limiting factor is the resistance of soft tissues.

Distraction osteogenesis is a treatment method that not only expands bony structures but also initiates a sequence of adaptive soft tissues changes termed distraction histogenesis.

Then a question can be made if segment reposition by distraction osteogenesis would allow larger movements than orthodontics only, i.e. beyond orthodontic limits.

From the orthodontic point of view it must be realized that no pre-surgical coordination of the entire dental arches like in conventional orthognathic surgery is made, but dental arches are aligned in segments. Preoperative orthodontics often includes preparation of spaces for osteotomies and postoperative uprighting of the tipped teeth.

Because of the change in the traditional orthognathic surgery close collaboration between surgeon and orthodontist is extremely important already in the planning phase of the treatment utilizing segmented therapy.

